# Misclassification of incident conditions using claims data: impact of varying the period used to exclude pre-existing disease

**DOI:** 10.1186/1471-2288-13-32

**Published:** 2013-03-06

**Authors:** Robert I Griffiths, Cynthia D O’Malley, Robert J Herbert, Mark D Danese

**Affiliations:** 1 Outcomes Insights, Inc., 340 North Westlake Blvd., Suite 200, Westlake Village, CA 91362, USA; 2 Johns Hopkins University School of Medicine, Baltimore, MD, USA; 3 Department of Primary Care Health Sciences, University of Oxford, Oxford, UK; 4 Amgen, Inc., South San Francisco, CA, USA; 5 Johns Hopkins Bloomberg School of Public Health, Baltimore, MD, USA

**Keywords:** Incidence, Prevalence, Misclassification, Look back, Medical claims, Medicare

## Abstract

**Background:**

Estimating the incidence of medical conditions using claims data often requires constructing a prevalence period that predates an event of interest, for instance the diagnosis of cancer, to exclude those with pre-existing conditions from the incidence risk set. Those conditions missed during the prevalence period may be misclassified as incident conditions (false positives) after the event of interest.

Using Medicare claims, we examined the impact of selecting shorter versus longer prevalence periods on the incidence and misclassification of 12 relatively common conditions in older persons.

**Methods:**

The source of data for this study was the National Cancer Institute’s Surveillance, Epidemiology, and End Results cancer registry linked to Medicare claims. Two cohorts of women were included: 33,731 diagnosed with breast cancer between 2000 and 2002, who had ≥ 36 months of Medicare eligibility prior to cancer, the event of interest; and 101,649 without cancer meeting the same Medicare eligibility criterion. Cancer patients were followed from 36 months before cancer diagnosis (prevalence period) up to 3 months after diagnosis (incidence period). Non-cancer patients were followed for up to 39 months after the beginning of Medicare eligibility. A sham date was inserted after 36 months to separate the prevalence and incidence periods. Using 36 months as the gold standard, the prevalence period was then shortened in 6-month increments to examine the impact on the number of conditions first detected during the incidence period.

**Results:**

In the breast cancer cohort, shortening the prevalence period from 36 to 6 months increased the incidence rates (per 1,000 patients) of all conditions; for example: hypertension 196 to 243; diabetes 34 to 76; chronic obstructive pulmonary disease 29 to 46; osteoarthritis 27 to 36; congestive heart failure 20 to 36; osteoporosis 22 to 29; and cerebrovascular disease 13 to 21. Shortening the prevalence period has less impact on those without cancer.

**Conclusions:**

Selecting a short prevalence period to rule out pre-existing conditions can, through misclassification, substantially inflate estimates of incident conditions. In incidence studies based on Medicare claims, selecting a prevalence period of ≥24 months balances the need to exclude pre-existing conditions with retaining the largest possible cohort.

## Background

Medicare administrative and claims data have many uses [1], including estimating the overall incidence of chronic and acute conditions [2-13], as well as the incidence of adverse events related to medical interventions [14-17] or within certain care settings [18]. Since information on medical history is not generally available in Medicare data - with some exceptions where claims are linked to other data sets [7,18] – researchers interested in estimating disease incidence, especially chronic disease incidence, often divide the observation period into two discrete intervals: a prevalence period, in which patients already diagnosed with the condition are identified and excluded from the incident condition risk set; and an incidence period, which is then searched for claims indicating the presence of previously undetected conditions [2,3,6,8,11,12,15]. In studies where one is interested in the incidence of conditions that follow a specific clinical event, such as the diagnosis of cancer, the onset of end-stage renal disease, or a medical or surgical intervention, the date of diagnosis or intervention typically is used to define the end of the prevalence period and the beginning of the incidence period [3,4,15]. Researchers then look backwards from that date a specific number of months or years to define the prevalence period, and either they require that all patients included in the study have at least that amount of time under observation prior to that date [3,15], or they use all of the data back to the beginning of the claims period [4]. In other cases, a “sham” index date may be selected to indicate the beginning of the incidence period following a pre-defined period of observation [2,6,8,11,12], for instance 12 months of Medicare eligibility.

**Figure 1:**
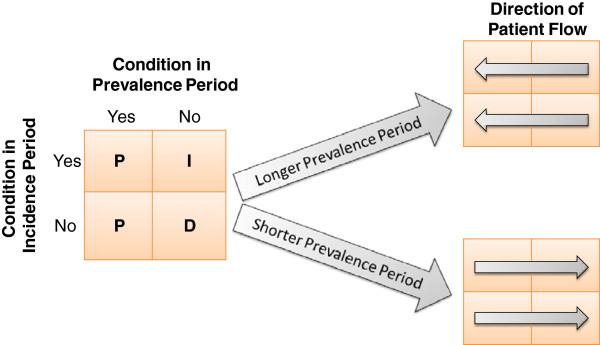
**Classification of conditions based on length of the prevalence period.** Figure describes the impact of changing the length of the prevalence period on the classification of a patient condition into one of four mutually exclusive groups: condition present in both prevalence and incidence periods; condition present in prevalence period only; condition present in incidence period only; and condition present in neither prevalence nor incidence periods. (Left-hand box) P indicates that that condition is classified as prevalent and is excluded from the risk set for incidence. I indicates the condition is incident. The incidence is calculated as I/I + D, where D is the count of patients without the condition in either the prevalence or incidence period. Increasing the prevalence period (upper right-hand box) results in more conditions being classified as prevalent. Patients with the condition present in the incidence period only can be reclassified as having the condition present in both the prevalence and incidence periods (upper arrow within upper right-hand box). Patients without the condition in either the prevalence or the incidence period can be reclassified as having the condition in the prevalence period only (lower arrow within upper right-hand box). Patients move in the opposite direction when the prevalence period is shortened (lower right-hand box).

Selecting the length of the prevalence period often entails a tradeoff between A) sample-size, which tends to be larger with shorter prevalence periods, especially when dates of diagnosis or intervention defining the end of the prevalence period are distributed throughout a block of data defined by a calendar period [3,4,15], and B) the ability to identify and exclude patients with pre-existing conditions from the incidence risk set. If one considers the entire observation period (prevalence period and incidence period combined), then each individual can fall into one of four mutually exclusive groups for each condition of interest: condition is present in both the incidence and prevalence periods; condition is present only in the incidence period; condition is present only in the prevalence period; and condition is present in neither the prevalence nor the incidence period. Lengthening the prevalence period may reassign patients: first, from the “neither” group to the “prevalence only” group; second, from the “incidence only” group to the “both” group. Shortening the prevalence period will have the opposite effect (Figure 1).

In theory, one could pick a sufficiently long prevalence period such that the “incidence only” group would include only true positives, assuming, of course, perfect sensitivity and specificity for the information used to identify the conditions (e.g., claims data). That is to say, additional lengthening of the prevalence period will not result in any additional reclassifying of “incidence only” patients as “both prevalence and incidence” patients, or in their removal from the incident risk set. Conversely, shortening the prevalence period from this point would add false positives to the true positives in the “incidence only” group. Adding false positives naturally would inflate incidence estimates. Furthermore, it could, in theory, increase type II error in studies of risk factors for the conditions, or studies on the outcomes of the conditions.

The objective of this study was to examine the impact of changing the length of the prevalence period on the observed incidence and misclassification of these conditions.

## Methods

### Data source

The source of data for this study was the National Cancer Institute’s (NCI) Surveillance, Epidemiology, and End Results (SEER) cancer registry linked to Medicare claims [19]. SEER contains cancer incidence and survival data from population-based cancer registries throughout the United States, presently covering approximately 28% of the population [20]. In SEER-Medicare, cancer registry data are linked to Medicare enrollment and claims data, which are available for 93% of those aged ≥ 65 years in the SEER registry [21]. Claims available in SEER-Medicare include hospital short- and long-stay, skilled nursing facility, physician/supplier, institutional outpatient, home health agency, and durable medical equipment. Our dataset also included a separately created 5% random sample of non-cancer patients from the Medicare program in the same catchment areas as those in the SEER program.

### Inclusion criteria

Two cohorts, cancer and non-cancer, were included in the study. The cancer cohort consisted of women diagnosed with breast cancer between January 1, 2000, and December 31, 2002, who had at least 36 months of Medicare Part A (hospital) and Part B (outpatient) fee-for-service coverage prior to the diagnosis of cancer. Medicare and other sources of administrative and claims data also are used to examine the epidemiology of disease in patients without cancer. Patterns of incidence misclassification could differ between those with cancer and others in the general population, due to shared risk factors and also detection bias associated with diagnostic work-up for cancer and cancer treatment. Thus, for comparison, we also included in this study a cohort of Medicare beneficiaries who had not been diagnosed with cancer. The non-cancer cohort consisted of all women in the sample who reached a total of at least 36 months of Medicare Part A and Part B fee-for-service coverage during the same time period (January 1, 2000, and December 31, 2002). Therefore, the calendar intervals defining the prevalence and incidence periods were identical in the cancer and non-cancer cohorts.

### Observation period

Patients in the cancer cohort were followed from 36 months before to up to three months after the date of diagnosis. Since SEER provides only the month and year of diagnosis, the first day of the month was used as the date of diagnosis. Patients in the non-cancer cohort were assigned a “sham” index date on the first day of the 37th month after the beginning of Medicare Part A and B coverage. The observation period was divided into two periods. The prevalence period consisted of 36 months prior to cancer diagnosis or the sham index date. The incidence period consisted of three months following cancer diagnosis or the sham index date.

### Patients and variables

Patients in both cohorts were described according to age, race/ethnicity, SEER region, and NCI Comorbidity Index score [22,23]. Additional variables from SEER, including cancer stage, grade, estrogen and progesterone receptor status, and year of diagnosis, were used to describe the cancer cohort.

International Classification of Diseases, 9th Edition, Clinical Modification (ICD-9-CM) codes [24] within Medicare claims were used to identify 12 common chronic and acute conditions in the elderly, consisting of diabetes, hypertension, congestive heart failure, chronic obstructive pulmonary disease (COPD), cerebrovascular disease, renal disease, osteoarthritis, myocardial infarction, depression, osteoporosis, liver disease, and hip fracture. (Table 1) Diagnoses recorded on claims for inpatient stays were counted at the time of their first occurrence. Diagnoses in outpatient facility and physician claims were assessed similarly to the NCI Comorbidity Index algorithm, which requires two diagnoses at least 30 days apart to identify a condition (taking the first occurrence as the date of onset).

**Table 1:** International classification of diseases, 9th edition, clinical modification (ICD-9-CM) codes used to identify conditions

**Condition**	**Code type**	**Codes**
**Cerebrovascular disease**	ICD-9-CM diagnosis	430-437.1, 437.3-438.xx
**Congestive heart failure**	ICD-9-CM diagnosis	428.xx, 398.91, 402.01, 402.11, 402.91, 404.01, 404.03, 404.11, 404.13, 404.91, 404.93
**Chronic obstructive pulmonary disease**	ICD-9-CM diagnosis	491.x-492.x, 496
**Depression**	ICD-9-CM diagnosis	296.2x, 296.3x, 298.0, 300.4, 309.0, 309.1, 309.28, 311
**Diabetes**	ICD-9-CM diagnosis	250.xx, 357.2, 362.0x, 366.41, 648.0x
**Hip fracture**	ICD-9-CM diagnosis	820.xx
**Hypertension**	ICD-9-CM diagnosis	401.xx-405.xx, 437.2
**Liver disease**	ICD-9-CM diagnosis	571.2, 571.4-571.49, 571.5, 571.6, 571.8, 571.9, 572.2-572.8, 456.0-456.21, V42.7
**Myocardial infarction**	ICD-9-CM diagnosis	410.xx, 411.0, 412.xx, 429.7x
**Osteoarthritis**	ICD-9-CM diagnosis	715.xx
**Osteoporosis**	ICD-9-CM diagnosis	733.xx
**Renal disease**	ICD-9-CM diagnosis	285.21, 403.xx-404.xx, 405.01, 405.11, 405.91, 458.21, 582.xx, 583.xx, 585, 586, 588.xx, 593.71-593.73, V42.0

### Analysis

For each condition, each patient in each cohort was assigned to one of four mutually exclusive groups based on when the condition was identified: in both the prevalence and incidence periods; in the prevalence period only; in the incidence period only; and in neither the prevalence nor the incidence period. The 36-month prevalence period was used to exclude previously diagnosed patients to permit the identification of the incident cases (and to calculate the incidence of each condition). This analysis was considered the “gold standard” for comparing other intervals for the prevalence period, with the incident patients considered to be “true positives”.

The length of the prevalence period was then reduced in 6-month increments from 30 months to only 6 months before cancer diagnosis or the sham date. Patients were reassigned to one of the four groups for each of the prevalence periods. Shortening the prevalence period meant that some patients in the prevalence only group might move to the neither group, and those in the prevalence and incidence group might move to the incidence only group. (Figure 1) For each prevalence period (30, 24, 18, 12, and 6 months), we recalculated the incidence and the number of patients added to the incidence only group as a result of shortening the prevalence period. Patients added to the incidence only group, as an artifact of shortening the prevalence period, were classified as false positives, and for each shorter prevalence period we calculated (A) the false positive fraction, which was defined as the number of false positives in the incidence period divided by the number of conditions identified in the “gold standard” 36-month prevalence period, and (B) the percent of all incident cases that were false positives. Since all patients classified as incident cases using a 36-month prevalence period also were classified as incident cases using shorter prevalence periods, the “sensitivity” of using shorter prevalence periods (probability of being classified as an incident condition using a shorter prevalence period given one was classified as an incident condition using the 36-month prevalence period) =1 for all the shorter prevalence periods. However, the specificity (probability of not being classified as an incident condition using a shorter prevalence period given one was not classified as an incident condition using the 36-month prevalence period) declines with shorter prevalence periods, causing the false positive fraction to increase and the positive predictive value to decrease.

The epidemiology of conditions, as well as patterns of misclassification, could differ between the cancer and non-cancer cohorts based on shared risk factors for conditions and cancer, and also that cancer is likely to result in the increased detection (detection bias) of pre-existing, but previously undiagnosed conditions. Therefore, these two cohorts were analyzed separately.

This study was conducted as part of a protocol submitted to Quorum Review Institutional Review Board. On November 10, 2011, Quorum granted a determination of exemption for this protocol, based on the fact that the information in the data files is recorded in such a manner that subjects cannot be identified either directly or through identifiers linked to them. The Quorum Review File # for the exemption determination is 26648.

## Results

There were 33,731 cancer and 101,649 non-cancer patients. Characteristics are described in Table 2. Based on the 36-month prevalence period, incidence proportions per 1,000 patients ranged from 196.3 for hypertension to 1.0 for liver disease in the cancer cohort, and from 13.1 for hypertension to 0.2 for liver and renal disease in the non-cancer cohort (Table 3). Prevalence proportions also were higher for cancer patients, although the differences between the two cohorts were not as large.

**Table 2:** Demographic characteristics of the breast cancer and non-cancer patient cohorts

		**Breast cancer**	**Non-cancer**
**Characteristic**		**patients**	**patients**
		**N = 33,731 (%)**	**N = 101,649 (%)**
**Age (years)**^**a**^	**≤ 70**	5,498 (16.3)	27,783 (27.3)
	**71-75**	9,618 (28.5)	29,883 (29.4)
	**76-80**	8,903 (26.4)	24,098 (23.7)
	**> 80**	9,712 (28.8)	19,885 (19.6)
**Race/ethnicity**	**White**	29,487 (87.4)	85,329 (83.9)
	**Black**	2,173 (6.4)	7,483 (7.4)
	**Asian/Other**	1,016 (3.0)	6,317 (6.2)
	**Hispanic**	1,055 (3.1)	2,520 (2.5)
**SEER Region**^**c**^	**Georgia (Atlanta/Rural Georgia)**	1,009 (3.0)	3,575 (3.5)
	**California**^**b**^	10,533 (31.2)	30,910 (30.4)
	**Connecticut**	2,121 (6.3)	6,380 (6.3)
	**Hawaii**	442 (1.3)	1,712 (1.7)
	**Iowa**	2,397 (7.1)	7,172 (7.1)
	**Kentucky**	2,889 (8.6)	8,680 (8.5)
	**Louisiana**	2,355 (7.0)	6,920 (6.8)
	**Michigan (Detroit)**	2,638 (7.8)	8,029 (7.9)
	**New Jersey**	5,950 (17.6)	15,961 (15.7)
	**New Mexico**	717 (2.1)	2,751 (2.7)
	**Utah**	851 (2.5)	3,006 (3.0)
	**Washington (Seattle/Puget Sound)**	1,829 (5.4)	4,665 (4.6)
**NCI Comorbidity score**^**d**^	**0**	27,193 (80.6)	76,804 (75.6)
	**1**	3,965 (11.8)	18,087 (17.8)
	**≥2**	2,573 (7.6)	6,758 (6.6)
**Year of diagnosis**	**2000**	11,433 (33.9)	N/A
	**2001**	11,432 (33.9)	
	**2002**	10,866 (32.2)	

^a^Age at diagnosis index date.

^b^California includes Los Angeles, San Francisco/Oakland, San Jose/Monterey, and Greater California.

^c^1,888 non-cancer patients had no region reported.

^d^Comorbidity index based on conditions identified in the 12 months prior to the diagnosis index date.

**Table 3:** Incidence and prevalence proportions of conditions in breast cancer and non-cancer cohorts

	**Incidence**^**a**^**proportion**		**Prevalence**^**a**^** proportion**	
**Condition**	**Breast cancer**	**Non-cancer**	**Breast cancer**	**Non-cancer**
**Cerebrovascular disease**	13.1	3.1	55.1	33.0
**Chronic obstructive pulmonary disease**	28.6	2.7	66.1	34.0
**Congestive heart failure**	19.8	2.8	76.9	32.1
**Depression**	14.2	1.5	35.1	21.1
**Diabetes**	33.5	3.4	91.1	46.8
**Hip fracture**	3.7	1.1	17.3	10.6
**Hypertension**	196.3	13.1	319.8	161.2
**Liver disease**	1.0	0.2	2.9	1.5
**Myocardial infarction**	12.7	2.0	34.8	23.1
**Osteoarthritis**	27.4	4.6	83.8	58.0
**Osteoporosis**	22.4	2.2	40.7	23.1
**Renal disease**	2.2	0.2	8.0	2.9

^a^ Both incidence and prevalence proportions reported as /1,000 patients at risk. Incidence period was 3 months after the date of cancer diagnosis (cancer patients) or sham date (non-cancer patients). Prevalence period was 36 months before the date of cancer diagnosis (cancer patients) or sham date (non-cancer patients).

**Figure 2:**
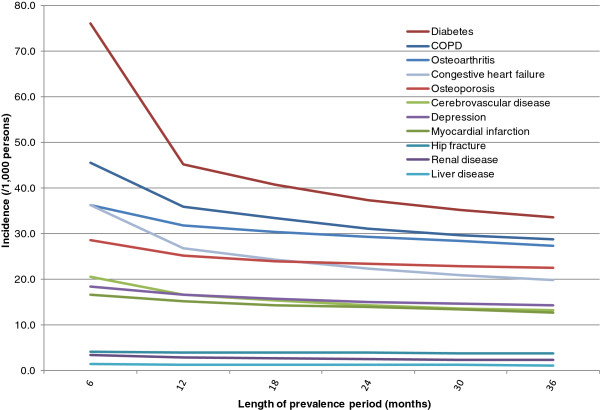
**Incidence of conditions by duration of the prevalence period – breast cancer cohort.** The prevalence period is the observation period used to exclude patients with the condition prior to cancer diagnosis. Hypertension is not shown to avoid compression of the vertical axis. Hypertension rates (/1,000) are as follows: 6 months 343.4; 12 months 239.4; 18 months 222.2; 24 months 211.9; 30 months 203.8; and 36 months 196.3.

**Figure 3:**
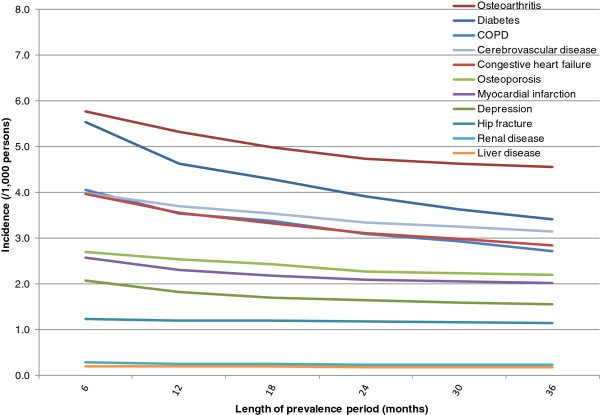
**Incidence of conditions by duration of the prevalence period – non-cancer cohort.** The prevalence period is the observation period used to exclude patients with the condition prior to sham index date. Hypertension is not shown to avoid compression of the vertical axis. Hypertension rates (/1,000) are as follows: 6 months 19.6; 12 months 16.9; 18 months 15.5; 24 months 14.5; 30 months 13.8; and 36 months 13.1.

The incidence of all conditions increased in both the cancer (Figure 2) and non-cancer (Figure 3) cohorts as the prevalence period was shortened in 6-month increments from 36 to 6 months. For example, in cancer most incidences increased as follows: hypertension 196 to 243; diabetes 34 to 76; COPD 29 to 46; osteoarthritis 27 to 36; congestive heart failure 20 to 36; osteoporosis 22 to 29; cerebrovascular disease 13 to 21; depression 14 to 18; and myocardial infarction 13 to 17. Patterns were similar in the cancer and non-cancer cohorts, with the biggest increases in incidence when the prevalence period was shortened from 12 months to 6 months.

**Figure 4:**
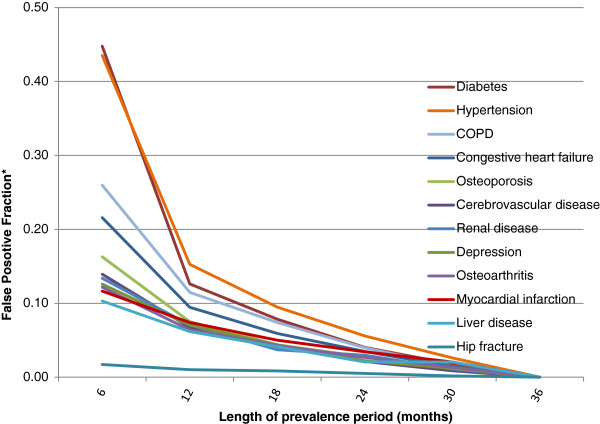
False Positive Fractions* – breast cancer cohort. A false positive fraction is defined as the number of false positives in the incidence period divided by the number of conditions identified in the “gold standard” 36-month prevalence period.

**Figure 5:**
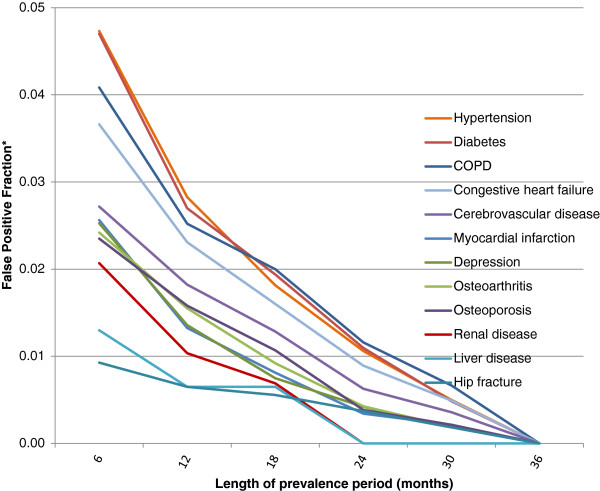
False Positive Fractions* – non-cancer cohort. A false positive fraction is defined as the number of false positives in the incidence period divided by the number of conditions identified in the “gold standard” 36-month prevalence period.

In the cancer cohort false positive fractions for the prevalence period of 6 months ranged from 0.44 for diabetes and hypertension to 0.02 for hip fracture and <0.01 for pancreatitis. False positive fractions declined rapidly between prevalence periods of 6 and 12 months, and more slowly thereafter. (Figure 4) Patterns of false positive fractions were similar, but an order of magnitude smaller, in the non-cancer cohort (Figure 5).

**Figure 6:**
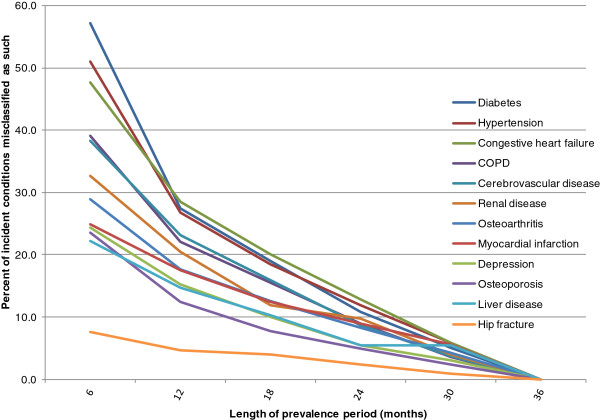
**Proportion of false positive incident conditions – breast cancer cohort.** The prevalence period is the observation period used to exclude patients with the condition prior to cancer diagnosis.

**Figure 7:**
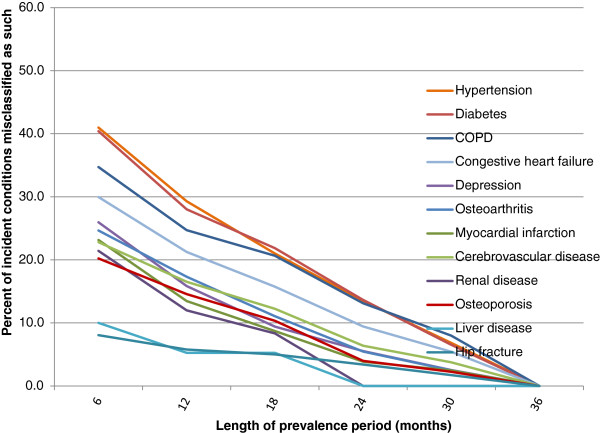
**Proportion of false positive incident conditions – non-cancer cohort.** The prevalence period is the observation period used to exclude patients with the condition prior to the sham diagnosis date.

In the cancer cohort, more than 50% of incident diabetes and hypertension cases identified using a 6-month prevalence period were misclassified as false positives (Figure 6), because they would have been excluded as prevalent cases had the prevalence period been 36 months. Based on a prevalence period of 6 months, false positive incident cases comprised more than 20% of the total incident cases for 11/12 conditions. The greatest decline in misclassification occurred when the prevalence period was lengthened from 6 to 12 months. Patterns of misclassification were similar in the non-cancer cohort, but misclassification occurred at lower rates (Figure 7).

## Discussion

Uses of Medicare administrative and claims data include estimating the incidence of acute and chronic medical conditions [2-13]. There is also considerable interest in using claims to identify adverse events related to treatment [14-17], and this interest may well strengthen with the availability of Medicare Part D oral drug data, and as the United States Food and Drug Administration explores new data structures and methodologies for identifying and analyzing adverse drug events [25]. Many such analyses require that the investigator define a prevalence period to exclude patients with pre-existing conditions from the incidence risk set [2,3,6,8,11,12,15]. In this study, we sought to understand how changing the duration of the prevalence period, and specifically shortening it from a “gold standard” of 36 months, would affect the incidence estimates for 12 chronic and acute conditions common among the elderly. We found that the number of false positive incident cases increased considerably as the length of the prevalence period was shortened. Using a 6-month prevalence period, false positives accounted for more than 50% of all incident hypertension and diabetes cases in the cancer cohort, and there were also high false-positive rates in the non-cancer cohort.

Our findings suggest that studies using relatively short prevalence periods to exclude pre-existing disease from the incident risk set may overestimate the incidence of the condition because the risk set includes false positive (undetected) prevalent cases. This may be especially problematic in studies that estimate trends in the annual incidence of conditions *where the prevalence also is increasing* - namely chronic conditions like diabetes, where we observed the greatest increase in false positive incident cases associated with shortening the prevalence period. To minimize potential misclassification when repeat acute conditions are classified as prevalent (false-negative), it is critical to use disease-specific algorithms to distinguish follow-up care from new events. For example, a six month lag between two hip fracture claims would be likely to represent two separate incident fractures rather than chronic care for a single fracture [26].

We constructed our cancer cohort from SEER-Medicare, and used the date of cancer diagnosis to divide the observation period into prevalence and incidence periods. Researchers using SEER-Medicare for epidemiologic studies typically require only 12 months of Medicare Part A and Part B coverage prior to cancer diagnosis to calculate an NCI Comorbidity Index [22,23]. In studies on the incidence of conditions, possibly including adverse-events related to cancer treatment where a comparator group is not included [15], the look-back period used to define the NCI Comorbidity Index also doubles as the prevalence period for excluding pre-existing conditions. Our findings show that with a 12-month prevalence period, false positives accounted for 20-30% of all incident diabetes, hypertension, congestive heart failure, COPD, cerebrovascular disease, and renal disease cases in cancer patients. Therefore, establishing a prevalence period based on the requirements for calculating the NCI Comorbidity Index could result in overestimating the incidence of conditions at the time of cancer diagnosis by up to 30%. Also, diluting the true positive incident cases with false positives could mask underlying associations between incident conditions and other variables. Consequently, it is advisable for researchers to consider longer prevalence periods if they are using SEER-Medicare to investigate the incidence of conditions, especially chronic conditions, that occur around the time of cancer diagnosis. However, this should be weighed against the loss of sample size that can result from requiring patients to have longer periods of Medicare Part A and B coverage prior to cancer diagnosis.

The differences between the cancer and the non-cancer cohort deserve comment. It is difficult to compare these populations directly because, in the cancer cohort, certain comorbid conditions may also be associated with cancer. Hence, one should not expect the same prevalence or incidence estimates in both cohorts, even with adjustment for age and other common confounders. Despite this, it is instructive to see how much larger the incidence rates are for most conditions in the cancer cohort (i.e., on the order of 3–15 times higher). Most likely, this is due to the diagnostic workup as part of the diagnosis of cancer and/or treatment planning – “detection bias” - suggesting that studies of cohorts undergoing a sentinel event (like breast cancer or myocardial infarction) may find additional comorbid conditions after the index event. Whether these should be considered as “baseline” comorbid conditions is something to be considered, and would depend on the condition and the importance of identifying the condition in the study.

Our study has several limitations. First, we defined a 36-month prevalence period as the gold standard for defining true positive incident cases. It is possible that had we used a longer prevalence period, some of the true positive incident cases would have been excluded from the risk set, having also appeared in the additional prevalence period. Extending the prevalence period can only reduce, not increase, the number of incident cases. Therefore, selecting a shorter rather than longer gold standard can only result in under-estimating the number of false positives. In this regard, our study may present conservative estimates of misclassification.

Second, we selected cohorts that only included patients with 36 months of Medicare Part A and B coverage prior to cancer diagnosis (cancer cohort) or the sham date (non-cancer cohort). This was necessary to identify true positive incident cases in a stable cohort. As discussed above, in reality, cohort sizes would increase as the required prevalence period gets shorter. This, in turn, could affect the incidence of the conditions we studied.

Third, we included only 12 conditions that are relatively common in older patients. Many are included in the NCI Comorbidity Index, and others are of interest as they could result from cancer interventions, or they could directly or indirectly influence cancer outcomes. In order for conditions to be defined as false positives in the incident analysis, they had to have been present in both the 36-month prevalent and incident periods, but then to drop out of the prevalent period as it became shorter. This may best describe chronic conditions with diagnoses that appear only infrequently in claims but then become relevant for medical decision-making around the time of cancer diagnosis. Patterns of misclassification for other conditions may differ from the ones we included in this study. Similarly, different definitions of these chronic conditions, i.e., the use of different ICD-9 codes or different thresholds for the number, timing, or type of claims, may give different results.

Fourth, we elected to divide the observation period into prevalence and incidence periods based on the date of cancer diagnosis. However, to the extent that diagnostic work-up for cancer begins prior to diagnosis, and that this results in increased detection of new conditions, changing the point at which the prevalence period ends and the incidence period begins could affect the incidence rates and the amount of misclassification. As with most studies based on SEER-Medicare, Medicare claims, or other sources of administrative and claims data, we did not include a “true gold standard” with perfect sensitivity and specificity for ascertaining the prevalences and incidences of the conditions. Finally, our cancer cohort included only women with breast cancer. It is possible that patterns of misclassification could differ in other types of cancers.

## Conclusions

Estimating the incidence of medical conditions using claims data often requires constructing a prevalence period that predates an event of interest to exclude those with pre-existing conditions from the incidence risk set. Our findings show that selecting a short prevalence period can, through misclassification, substantially inflate incidence estimates. This is especially problematic with chronic conditions like diabetes. Selecting longer prevalence periods will mitigate this problem. Based on our findings, we recommend a prevalence period of ≥24 months, especially if the focus of the incidence analysis is on estimating the incidence of chronic conditions that may appear less frequently in Medicare claims. However, this decision should be weighed against the associated loss of sample size and the need to include patients of younger age.

## Competing interests

Robert I. Griffiths, Robert J. Herbert, and Mark D. Danese work for Outcomes Insights, Inc. Outcomes Insights has received funding from Amgen, Inc. to conduct research on the epidemiology of chronic and acute conditions in cancer. Cynthia D. O’Malley is an employee and shareholder in Amgen, Inc.

## Authors’ contributions

RG and MD acquired the data. RG, MD, and CO’M developed the initial analysis plan. RH did the programming. All authors reviewed the findings. RG wrote the initial version of the manuscript, and RH created all tables and figures. All authors contributed to the revisions of the manuscript. All authors read and approved the final manuscript.

## Pre-publication history

The pre-publication history for this paper can be accessed here:

http://www.biomedcentral.com/1471-2288/13/32/prepub
